# Expression and Genetic Effects of GLI Pathogenesis-Related 1 Gene on Backfat Thickness in Pigs

**DOI:** 10.3390/genes13081448

**Published:** 2022-08-14

**Authors:** Xin Liu, Hanmei Li, Longchao Zhang, Ligang Wang, Lixian Wang

**Affiliations:** 1 Institute of Animal Science, Chinese Academy of Agricultural Sciences, Beijing 100193, China; 2 Hebei Normal University of Science & Technology, Qinhuangdao 066600, China

**Keywords:** *GLIPR1*, pig, backfat thickness, expression, genetic effects

## Abstract

Backfat thickness (BFT) is an important carcass composition trait and regarded as a breeding focus. Our initial transcriptome analysis of pig BFT identified GLI pathogenesis-related 1 (*GLIPR1*) as one of the promising candidate genes. This study was conducted to identify the expression profiles, polymorphisms, and genetic effects of the *GLIPR1* gene on BFT in pigs. The expression of the *GLIPR1* gene existed in every detected tissue, and there was a significantly higher expression in spleen and adipose tissue than others (*p* < 0.05). At the different ages of pig, the expression of the *GLIPR1* gene was low at an early age, increased with growth, and reached the highest level at 180 days. Genetic polymorphism analysis was detected in 553 individuals of the Large White × Minzhu F2 population. Four SNPs in the promoter significantly associated with 6–7 rib BFT (*p* < 0.05) were predicted to alter the transcription factor binding sites (TFBS), and the mutations of g.38758089 T>G and g.38758114 G>C were predicted to change the TFs associated with the regulation of adipogenesis. Haplotypes were formed by the detected SNPs, and one block showed a strong association with BFT (*p* < 0.05). In summary, our results indicate that the expression profiles and genetic variants of *GLIPR1* affected the BFT of pigs. To our knowledge, this study is the first to demonstrate the biological function and genetic effects of the *GLIPR1* gene on the BFT of pig and provide genetic markers to optimize breeding for BFT in pigs.

## 1. Introduction

Pork occupies a large part of the meat market. In order to meet consumer demand, reduced fat and increased lean meat content are sought in pig production. Backfat thickness (BFT) is an important carcass composition trait and influences growth, which is why it is regarded as a breeding focus [1,2,3]. Due to the importance of BFT, it has been extensively studied, and some important regulatory genes have been found [4,5,6]. GLI pathogenesis-related 1 (*GLIPR1*) was identified as a candidate gene by transcriptome analysis of pig BFT in our previous study [7].

GLIPR1 is also called RTVP1, belonging to the GAP protein superfamily [8], found early in human gliomas as a proto-oncogene [9]. The open reading frame of human *GLIPR1* contains 926 bp and encodes a polypeptide consisting of 266 amino-acid residues. *GLIPR1* has been found to affect various tumors by its expression change [10,11]. Overexpression of *GLIPR1* appears to enhance proliferation in glioblastoma, Wilm’s tumor, acute myeloid leukemia, etc. [12,13]. However, a reduction in *GLIPR1* expression inhibits and suppresses prostate cancer, lung and bladder cancer, and some other tumors [14,15]. It is indicated that *GLIPR1* is necessary for cell growth, proliferation, and pyroptosis [16]. The function of the *GLIPR1* gene was queried on the GeneCards (https://www.genecards.org/) (accessed on 5 February 2022), and we found this gene was involved in the peroxisome proliferator-activated receptor α (PPARα) pathway, which regulates lipid metabolism. PPARα is considered a transcriptional regulator of lipid metabolism, involved in fatty-acid transport and fatty-acid β-oxidation [17], and it is also recognized to be a target gene therapy for obesity and dyslipidemia for its effects on energy metabolism in adipocytes and activation in WAT [18]. We concluded that *GLIPR1* could regulate the fat formation through interaction with *PPARα*.

However, compared to the large number of studies in human disease, little information is available regarding *GLIPR1* in pigs. Our previous study revealed that different expressions of *GLIPR1* could regulate the backfat thickness of pigs, and this gene was located in the lipid related pathway by functional annotation. The objective of this study was to preliminarily clarify the function and genetic effects of *GLIPR1* in pig. In this study, we detected the expression profiles in various tissues and throughout the growth stages, and we further explored the polymorphisms and genetic effects of *GLIPR1* on pig BFT.

## 2. Materials and Methods

### 2.1. Ethics Statements

All methods and procedures in the study were carried out according to the standard guidelines on experimental animals, which were established by the Institute of Animal Sciences, Chinese Academy of Agricultural Sciences (IAS, CAAS).

### 2.2. Animals and Sample Collection

Large White pigs, selected from the pig farm of IAS, were used for the expression detection of *GLIPR1*. The three pigs of each age were slaughtered, and backfat samples were collected at ages 3 days, 60 days, 120 days, 150 days, and 180 days. Three 180 day old pigs were slaughtered, and tissue samples were rapidly collected from the backfat, longissimus muscle, heart, liver, lung, and kidney. All of the samples were prepared for mRNA expression detection and stored at −80 °C until use. The 553 pigs for single-nucleotide polymorphism (SNP) detection and association analysis were selected from the Large White × Minzhu F2 generation resource population, which was constructed and described in our previous studies [19]. All pigs were slaughtered at the age of 240 ± 7 days. The slaughter weight was measured, and BFT (6–7 ribs) was determined on each carcass using a Vernier caliper. The ear marginal tissues of individuals were collected for DNA isolation.

### 2.3. Genomic DNA and RNA Isolation

Genomic DNA exaction was performed using the traditional phenol–chloroform method. The quantity and quality of extracted DNA were evaluated by 1% agarose gel electrophoresis and then stored at −20 °C in a refrigerator. Total RNA was exacted according to the instructions of the RNAprep pure Tissue Kit (TIANGEN, DP431, Beijing, China). The concentration and the purity were measured by Nanodrop spectrophotometer (Thermo Scientific, Waltham, MA, USA), and the RNA integrity was determined by Agilent 2100, bioanalyzed (Agilent Technologies, Santa Clara, CA, USA), and then stored at −80 °C. First-strand cDNA was synthesized by reverse transcription using 1 μg of total RNA and the PrimeScriptTM RT reagent kit (TakaRa, Shiga, Japan) according to the manufacturer’s protocol; cDNAs were stored at −20 °C until use.

### 2.4. Expression Detection and Analysis of GLIPR1 Gene

To investigate the comparative levels of the expression of *GLIPR1* in different tissues, we extracted total RNA from various tissues, including heart, liver, spleen, hypothalamus, muscle tissue, and backfat. To compare the expression of *GLIPR1* in backfat among different ages of pig, total RNA was extracted from backfat samples of pigs at different ages. The TB GreenTM Premix Ex TaqTM (TaKaRa, Japan) was used to detect gene relative expression according to the instructions. SYBR Green Real-Time quantitative PCR was performed on an ABI 7500 instrument (Applied Biosystems, Inc., Foster City, CA, USA) to determine the expression level of *GLIPR1*, and *GAPDH* was used as the endogenous control gene. The mRNA reference sequences of *GLIPR1* and *GAPDH* were XM_021092042.1 and NM_001206359.1, respectively, which were obtained from NCBI (National Center for Biotechnology Information). The primers of *GLIPR1* and *GAPDH* (shown in Table 1) were designed by Primer Express 3.0 (Applied Biosystems). All primers were synthesized by Invitrogen trade limited company (Shanghai). The reaction system of 20 μL included 10 μL of 2 × TB Green Premix Ex Taq, 0.4 μL each of upstream and downstream primers (10 μmol/L), 0.4 μL of 50 × ROX Reference Dye II, 2 μL of cDNA, and 6.8 μL of ddH_2_O. The reaction procedure was as follows: 30 s pre-denaturation at 95 °C, then 40 cycles of 5 s denaturation at 95 °C and 34 s annealing at 60 °C, before finally obtaining the melting curve through 15 s at 95 °C, 1 min at 60 °C, and 15 s at 95 °C. The gene expression levels were quantified using the comparative 2^−^^ΔΔCT^ method [20], and the expression differences were analyzed using the ANOVA method in SAS 9.2 software (SAS INSTITUTE Inc., Cary, NC, USA).

### 2.5. SNP Identification and Association Analysis

On the basis of the genomic sequence of pig *GLIPR1* (GenBank accession number: NC_010447.5), we identified the SNPs located in the promoter regions of GLIPR1 using PCR amplification and sequencing for 553 F2 resource population individuals. The PCR primers were designed using the Primer 5.0 software (Premier Biosoft International, Palo Alto, CA, USA) (shown in Table 1). The reaction system of 25 μL included 2.5 μL of 10 × PCR buffer (including 15 mM Mg^2+^), 1 μL of dNTPs (2.5 mM each), 0.5 μL of each primer (10 μM), 1 μL of genomic DNA, 0.5 μL of Taq DNA polymerase (5 units/μL) (TaKaRa, Japan), and ddH_2_O made up the final volume. The reaction procedure was as follows: 94 °C for 5 min, followed by 35 cycles at 95 °C for 30 s, 1 min at annealing temperature, 72 °C for 30 s, and final extension at 72 °C for 10 min. PCR products were detected by 1.5% agarose gel electrophoresis.

As for the SNPs in the promoter region, we predicted whether these mutations altered the transcription factor binding sites (TFBS) using JASPAR software (https://jaspar.genereg.net/) (accessed on 5 February 2022). Genotypes and population genetic characteristics, including genotype frequency, allele frequency, polymorphic information content (PIC), gene heterozygosity (He), and the Hardy–Weinberg equilibrium test of polymorphic loci, were calculated. The association between genotypes and BFT was performed using the GLM procedure of SAS, as follows:

Yij = μ + Gi + bW + eij,

where Yij is the phenotypic observation, μ is the overall mean, Gi is the fixed effect of genotype, W is the covariate of slaughter weight, b is the covariate regression coefficient, and eij is the random error. The data are presented as the mean ± standard error, and significant differences were considered at *p* < 0.05.

### 2.6. Linkage Disequilibrium (LD) and Haplotype Analysis

Haploview 4.1 (Broad Insititute, Cambridge, MA, USA) was used to perform the LD analysis among the identified SNPs and constructed blocks. Then, Package Haplo.stats was used to perform association analysis between haplotypes and BFT in R.

## 3. Results

### 3.1. Tissue Expression Profiles of GLIPR1

**Figure 1:**
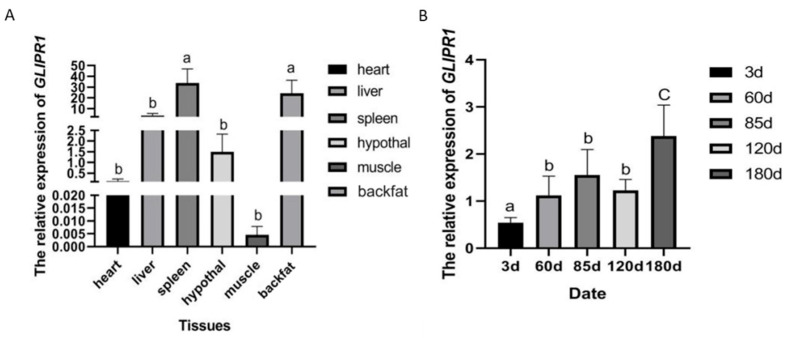
Comparison of relative expression of *GLIPR1* gene in different tissues of 180 day old pigs (**A**) and in backfat tissues among different growth ages of Large White pigs (**B**). Different letters indicate significant differences among the expression levels of *GLIPR1* gene at *p* < 0.05. Three biological replicates of each set were performed for qPCR.

Comparative analysis of *GLIPR1* expression was performed in six different tissues via real-time quantitative PCR (qPCR). The analysis showed that the levels of *GLIPR1* mRNA expression differed considerably among tissues. The highest relative expression was found in spleen and backfat (*p* < 0.05), and the lowest expression was found in muscle (*p* < 0.05) (Figure 1A). The relative expression of *GLIPR1* in backfat was examined in different ages of pigs by qPCR, and the results showed that the expression level of *GLIPR1* was low in the early growth stage of pig, then increased, and remained at a high expression in the middle and late stages. The expression level of *GLIPR1* in BFT of 3 day old pigs was significantly lower than other ages (*p* < 0.05), and the expression was highest in 180 day old pigs, which was significantly higher than that in the other ages of pigs (*p* < 0.05) (Figure 1B).

### 3.2. SNP Identification and Association with BFT

**Figure 2:**
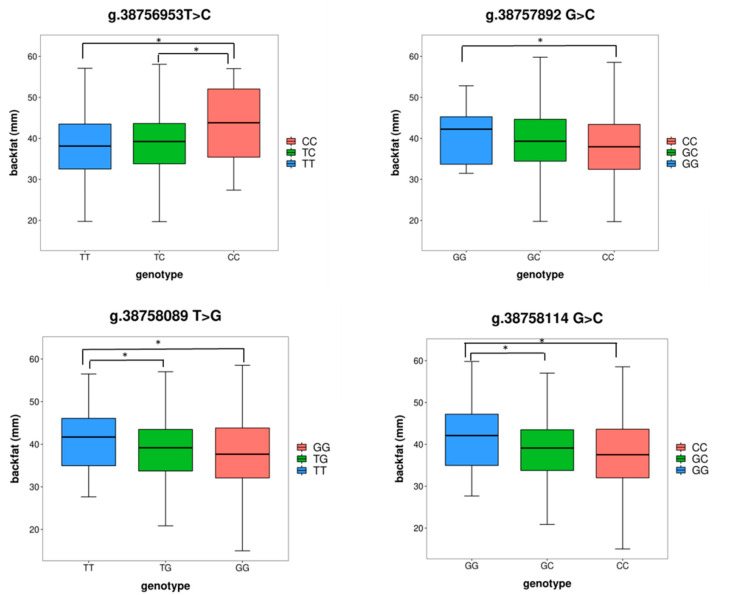
Significant association analysis between SNPs and BFT. * Significant difference in the association analysis between two genotypes and 6–7 rib backfat thickness at *p* < 0.05.

Since the mutations of promoter location may play an important role in the regulation of gene expression, the promoter region was first selected as the research object in this study. SNPs in the promoter regions of *GLIPR1* were identified for 553 individuals. A total of 33 SNPs in promoter regions were detected. We performed association analyses between these SNPs and 6–7 rib BFT in the F2 resource population, along with bioinformatics analysis. The results showed that four SNPs in promoter regions were not only significantly associated with BFT but also predicted at TFBS, which caused changes in transcription factors (TFs) (shown in Figure 2 and Table 2).

Genotyping of the identified SNPs significantly associated with BFT in the F2 resource population was performed, and the analysis of the genotype frequency and population genetic characteristics is shown in Table 3. The diversity parameters showed that the He values ranged from 0.2818 to 0.4050, and the PIC values ranged from 0.2421 to 0.3230. According to these data, all SNPs, except g.38757892 G>C, reached a medium level of genetic diversity (0.25 < PIC < 0.5).

### 3.3. Haplotype Analysis of GLIPR1 Gene

**Figure 3:**
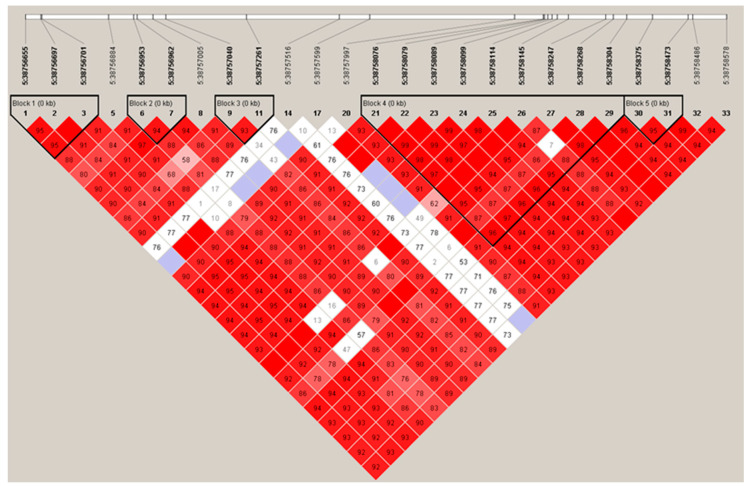
Linkage disequilibrium (LD) analysis of SNPs on promoter of *GLIPR1*; *r*^2^ is the correlation coefficient between the two loci.

LD analysis was performed among the 33 SNPs in promoter regions. We found five haplotype blocks in promoter regions (D’ = 0.86–0.95, Figure 3).

In the promoter regions of *GLIPR1*, the five blocks were correlated with BFT. Only block 2 had significant associations with BFT (*p* = 0.00143), while the other four blocks did not achieve significant correlations (*p* > 0.05). Block 2 had three haplotypes, among which the TA haplotype was significantly associated with BFT (*p* < 0.05). However, some haplotypes, including in blocks 1, 4, and 5, showed a strong association with BFT. The TAG haplotype in block 1 and GA haplotype in block 5 had significant negative effects (*p* < 0.05). The CCT haplotype in block 1, the TATCGACCG haplotype in block 4, and the AG haplotype in block 5 showed significant positive effects (*p* < 0.05) (Table 4).

## 4. Discussion

The BFT of pig has been a research hotspot for a long time because of its significance in pig breeding and production. With the development of omics technology and the reduction in chip and sequencing costs, there have been many studies on the important economic traits of pigs using omics methods, including BFT, and many new and interesting genes or mutations have been discovered [21,22,23]. In our previous study on the transcriptome analysis of pig BFT, the *GLIPR1* gene was identified as a candidate gene by analyzing the differentially expressed genes, comparing the high-BFT group with the low-BFT group, and finding its biological function related to adipogenesis [7]. In this study, further functional verification of the *GLIPR1* gene on the fat of pig was performed. The expression profiles, polymorphisms, and genetic effects were determined.

The expression of *GLIPR1* was detected among six tissues in 180 day old pigs. Although *GLIPR1* mRNA was present in all tissues, the levels varied considerably, with a higher expression observed in spleen and backfat and the lowest expression observed in muscle. Our results were basically consistent with those presented by NCBI (https://www.ncbi.nlm.nih.gov/gene/100523551) (accessed on 5 February 2022), which summarizes the detection results of this gene expression in the related literature [24]. The expression of *GLIPR1* in different growth ages showed that this gene had low expression at an early age and high expression at a late growth age, which revealed the regulation and action mode of this gene on adipogenesis in pigs. Research has reported that *GLIPR1* can affect cell-cycle regulation, and its changes in expression can regulate cell proliferation and apoptosis [12,25]. We hypothesize that *GLIPR1* may affect adipocyte differentiation and regulate adipogenesis in pig. At present, there is no report on the expression regulation of the *GLIPR1* gene on fat deposition in pigs; hence, this study provides some basis for further research.

According to the quantitative trait locus mapping on the PigQTL database (https://www.animalgenome.org/cgi-bin/QTLdb/SS/index) (accessed on 5 February 2022), the location of the *GLIPR1* gene on chromosome 5 is in the significant effect regions of backfat at the tenth rib [26] and backfat at the last lumbar [27]. It can be inferred that *GLIPR1* may play an important role in BFT. SNPs are genetic variations of single base pairs occurring in the genomic sequence impacting gene expression activities, amino-acid substitution, and dysfunction [28]. The occurrence and frequency of SNPs have contributed greatly to the assessment of genetic diversity and the genetic variation of complex quantitative traits [29,30]. Therefore, we detected the polymorphism of the *GLIPR1* gene and analyzed the association between the mutations and 6–7 rib BFT. The SNPs, which were significantly associated with BFT, can be genetic markers for pig breeding. The results which we obtained in the previous study showed that *GLIPR1* gene regulated fat deposition of pig through the differential expression. The mutations of promoter location may play an important role in the regulation of gene expression; hence, we first selected the promoter region for SNP polymorphism study. Among these significant associated mutations, four SNPs in promoter regions were predicted at TFBS, and their mutations lead to changes in binding TFs. TFs commonly control the gene expression through binding to specific sequence elements, and the regular regions [31] and the disruption of TFBs will show phenotypic diversity [32]. In our study, we found that four SNPs in the promoter altered TFBs, and some of them were associated with adipogenesis. The allele C of g.38758114 G>C created a TF NR2C2. *NR2C2*, known as a testicular receptor (tr4), is reported to regulate lipogenic gene expression, which is inactivated by the 5′-AMP-activated protein kinase (AMPK) and has been shown to play a potentially important role in insulin sensitivity [33]. The allele of G of g.38758089 T>G caused the disappearance of NFATC2. NFATC2 has been reported to regulate adipocyte differentiation by interacting with the transcription factor CCAAT/enhancer binding protein (C/EBP) and induce adipogenesis, as well as in obese mice [34,35].

Haplotypes formed by the SNPs have important implications for identifying associations with complex traits [36]. After haplotype and association analysis on *GLIPR1*, we obtained one block and some haplotypes significantly correlated with BFT. The significantly associated block in the promoter region contained the SNP g.38756953 T>G, which was also significantly correlated with BFT and predicted to cause TF change. The identified strongly significantly associated SNPs and haplotypes could be used as markers to optimize the selection and breeding of the BFT trait in pigs.

By studying genetic polymorphisms of the promoter region of *GLIPR1*, we obtained four potentially candidate SNPs related to BFT in pigs. However, this study had several limitations. The variants in other regions of this gene, including exons, introns, and UTR-regulated regions, as well as its surrounding regions, need more comprehensive studies in subsequent work.

## 5. Conclusions

Overall, our results indicate that the expression profiles and genetic variants of *GLIPR1* affected the BFT trait of pigs. This study is the first to demonstrate that the *GLIPR1* gene has a significant association with the BFT of pigs, preliminarily showing the biological effect of the *GLIPR1* gene on the subcutaneous fat of pig and providing genetic markers to optimize breeding for BFT in pigs.

## Figures and Tables

**Table 1:** Real-time quantitative PCR primes of *GLIPR1* and *GAPDH* and promoter primers of *GLIPR1.*

Primer Name	Primer F/R (5′–3′)
*GLIPR1*-F	CAACAAGCTCCGATCAGAAGTG
*GLIPR1*-R	GGGTGTAGCCTGTAGGGTGACT
*GAPDH*-F	TGGAGGCAAGGACCTAAAAGC
*GAPDH*-R	TCCACCACCCTGTTGCTGTAG
*PRIMER1*-F	AAGAAACTGAGCGGGCAAGG
*PRIMER1*-R	AGTGAGGTCAGGGATCAAAC
*PRIMER2*-F	ATACTTTACCAAAATGCTGC
*PRIMER2*-R	AACCCTCTTGAGTGATCTGC
*PRIMER3*-F	GTATGAGCAGATCACTCAAG
*PRIMER3*-R	AAAGCCAGTAGACCTAAAAG

**Table 2:** Changes of TFs caused by SNPs in promoter regions.

Location	Sequence	TF
**g.38756593 T>C**	CGGTCATATCTGAG**T**CTATGCATGCCTCTC *	
	CGGTCATATCTGAG**C**CTATGCATGCCTCTC	RHOXF1
**g.38757892 G>C**	CAGATCACTCAAGA**G**GGTTTTTCTCTTGTT	NKX2–4, NKX2–8
	CAGATCACTCAAGA**C**GGTTTTTCTCTTGTT	
**g.38758089 T>G**	ATGCACCTCTGCTA**T**TTTCCCTGGCAACAG	NFATC3, NFATC2
	ATGCACCTCTGCTA**G**TTTCCCTGGCAACAG	NFATC3
**g.38758114 G>C**	AACAGATGAGCCAA**G**CTGACTTCAATTCC	NFIX
	AACAGATGAGCCAA**C**CTGACTTCAATTCC	NFIX, NR2C2

* The identified SNPs are highlighted in bold.

**Table 3:** Population genetic characteristics of SNPs of *GLIPR1.*

Polymorphism Site	Genotype Frequency			Gene Frequency		χ^2^	PIC	He
**g.38756953 T>C**	TT	TC	CC	T	C	0.4508	0.2743	0.3282
	0.6243	0.3376	0.0381	0.7931	0.2069			
**g.38757892 G>C**	GG	GA	AA	G	A	26.8386	0.2421	0.2818
	0.0599	0.2196	0.7205	0.1697	0.8303			
**g.38758089 T>G**	TT	TG	GG	T	G	3.5656	0.3230	0.4050
	0.0958	0.3725	0.5361	0.2821	0.7179			
**g.38758114 G>C**	AA	AG	GG	A	G	3.5656	0.3230	0.4050
	0.0958	0.3725	0.5316	0.2821	0.7179			

**Table 4:** Correlation analysis between haplotype of *GLIPR1* promoter and backfat thickness.

Block	Haplotype	Haplotype Frequency	Haplotype Score	*p*-Value	Total *p*-Value
**Block 1**	TAG	0.70877	−2.25228	0.0243	0.1263
	TCT	0.01094	−0.31862	0.75002	
	CAG	0.00913	0.66759	0.5044	
	CCT	0.27116	2.23729	0.02527	
**Block 2**	TA	0.5125	−3.37577	0.00074	0.00143
	CA	0.20344	1.83631	0.06631	
	TG	0.2806	1.84942	0.0644	
**Block 3**	GG	0.22531	−0.91245	0.36153	0.09193
	AG	0.49587	−0.8357	0.40332	
	AA	0.27448	1.90887	0.05628	
**Block 4**	CGGTCAGTA	0.05715	−1.62615	0.10392	0.12506
	CGGTCTGTA	0.00861	−1.462	0.14374	
	CGGTCAGCA	0.58356	−1.33194	0.18288	
	CGGTCTGCA	0.04678	−0.60537	0.54493	
	CGGTCACCG	0.00482	−0.07727	0.93841	
	TATCGACCG	0.26571	2.10239	0.03552	
**Block 5**	GA	0.70004	−2.40065	0.01637	0.09626
	GG	0.0129	0.01944	0.98449	
	AA	0.00917	0.92225	0.3564	
	AG	0.27789	2.28996	0.02202	

## Data Availability

Not applicable.
